# Comparative psychophysics of Western honey bee (*Apis mellifera*) and stingless bee (*Tetragonula carbonaria*) colour purity and intensity perception

**DOI:** 10.1007/s00359-022-01581-y

**Published:** 2022-10-21

**Authors:** Sebastian Koethe, Lara Reinartz, Tim A. Heard, Jair E. Garcia, Adrian G. Dyer, Klaus Lunau

**Affiliations:** 1 Institute of Sensory Ecology, Heinrich-Heine-University Düsseldorf, 40225 Düsseldorf, Germany; 2 Sugarbag Bees, West End, QLD Australia; 3 School of Media and Communication, RMIT University, Building 5.2.36, City Campus, GPO Box 2476, Melbourne, VIC 3001 Australia; 4 Department of Physiology, Monash University, Melbourne, 3800 Australia; 5 Institute of Developmental Biology and Neurobiology, Johannes Gutenberg Universität, Mainz, Germany

**Keywords:** Stingless bees, Colour vision, Spectral purity, Colour intensity, Colour preference

## Abstract

Bees play a vital role as pollinators worldwide and have influenced how flower colour signals have evolved. The Western honey bee, *Apis mellifera* (Apini), and the Buff-tailed bumble bee, *Bombus terrestris* (Bombini) are well-studied model species with regard to their sensory physiology and pollination capacity, although currently far less is known about stingless bees (Meliponini) that are common in pantropical regions. We conducted comparative experiments with two highly eusocial bee species, the Western honey bee, *A. mellifera*, and the Australian stingless bee, *Tetragonula carbonaria*, to understand their colour preferences considering fine-scaled stimuli specifically designed for testing bee colour vision. We employed stimuli made of pigment powders to allow manipulation of single colour parameters including spectral purity (saturation) or colour intensity (brightness) of a blue colour (hue) for which both species have previously shown innate preferences. Both *A. mellifera* and *T. carbonaria* demonstrated a significant preference for spectrally purer colour stimuli, although this preference is more pronounced in honey bees than in stingless bees. When all other colour cues were tightly controlled, honey bees receiving absolute conditioning demonstrated a capacity to learn a high-intensity stimulus significant from chance expectation demonstrating some capacity of plasticity for this dimension of colour perception. However, honey bees failed to learn low-intensity stimuli, and *T. carbonaria* was insensitive to stimulus intensity as a cue. These comparative findings suggest that there may be some common roots underpinning colour perception in bee pollinators and how they interact with flowers, although species-specific differences do exist.

**Supplementary Information:**

The online version contains supplementary material available at 10.1007/s00359-022-01581-y.

## Introduction

Flower-visiting animals may develop different strategies to detect and exploit food sources. Most bees searching for food sources are limited by the distance between food source and hive, requiring efficient solutions (Visscher and Seeley 1982; Beekman and Ratnieks 2000; Greenleaf et al. 2007). Among bees, different behaviours or physiological mechanisms to locate food sources have evolved to facilitate the collection of floral rewards (Dornhaus et al. 2006; Dyer et al. 2008; Heard 2016).

Flower constancy is known for several bee species and is based on a bee’s fidelity towards a specific flower type for a period of time (Free 1963; Heinrich 1979; Wells and Wells 1983; Ramalho et al. 1994; Hill et al. 1997; Slaa et al. 1998). Thus bees may increase their efficiency by visiting the same type of rewarding flowers to help ensure known reward quality or quantity (Grant 1950; Free 1963; Hill et al. 1997; Chittka et al. 1999). Nonetheless, flower constancy may have the disadvantage that workers are less flexible and may ignore alternative rewarding flowers when resources change (Chittka et al. 1997; Dyer et al. 2014). Floral colour is assumed to have a strong impact on flower constancy in honey bees (Hill et al. 1997; Banschbach 1994; Gegear and Laverty 2004).

Honey bees are able to make very fine colour discriminations (von Helversen 1972; Dyer and Neumeyer 2005; Papiorek et al. 2013), but have coarse spatial acuity for colour stimuli (Giurfa et al. 1996), while bumble bees have more coarse colour discrimination but have a higher visual acuity to find rewarding flower resources (Dyer et al. 2008; Morawetz and Spaethe 2012). Probably, the trade-off between colour discrimination and colour detection evolved based on the respective foraging behaviour. Bumble bees forage on widely dispersed flower patches, while honey bees usually visit mass-flowering resources due to recruitment by nestmates (Dornhaus and Chittka 1999, 2004; Heinrich 2004). The higher visual acuity of bumblebees helps with the detection of small or sparse resources and is less advantageous in habitats with abundant resources. Furthermore, the waggle dance of honey bees provides no gain in patchy habitats, where individual target detection is more advantageous (Dornhaus and Chittka 1999, 2004; Sherman and Visscher 2002). Thus it is plausible that bees from different environmental conditions have different ways of processing colour signals, although only a few comparative studies exist.

Many studies have analysed colour preferences in Western honey bees and have concluded that colour stimuli with a “blue” hue (UV-blue, blue, blue-green) are preferred by Western honey bees and that the blue contrast interferes with shape learning (Menzel 1967; Giurfa et al. 1995; Zhang et al. 1995; Morawetz et al. 2013).

In a series of experiments, Rohde et al. (2013) trained Western honey bees and bumble bees to a particular colour stimulus and then presented in a subsequent test two additional stimuli, one with higher and another with lower spectral purity. Both bee species exhibited a significant preference towards the stimulus with a higher degree of spectral purity, rather than the actual trained colour stimulus. These findings correspond to earlier results with bumble bees, and lead to the assumption that spectral purity is an important parameter for colour choice in bumble bees and honey bees (Lunau 1990; Lunau et al. 1996), and potentially bee colour perception in general since the spectral spacing of photoreceptors in all tested bees is phylogenetically ancient and underpins colour perception (Briscoe and Chittka 2001). If evidence of a saturation preference was consistent for other bee species, it may help explaining how bees find and choose flowers in a way that could explain flower community assemble (Kantsa et al. 2017).

The influence of colour preferences on how stingless bees may choose flowers is still unclear. Brazilian stingless bees of the genus *Melipona* showed preferences for specific colour hues (yellow and UV-blue) while choices were not significantly influenced by either the intensity or spectral purity of stimuli (Koethe et al. 2016). In an experiment by Dyer et al. (2016a) innate preferences of the Australian stingless bee *Tetragonula carbonaria* were analysed by employing broadband colour stimuli from different regions of colour space, and like in honey bees, stimuli from the blue and blue-green regions of colour space were preferred. Furthermore, a combination of green contrast and spectral purity seemed to influence the worker’s preferences (Dyer et al. 2016a), although spectral purity as a single factor was not a significant factor explaining the observed behaviour. In recent research on the colour preferences for a range of stimuli in other Australian native bees, *Lasioglossum* (*Chilalictus*) *lanarium* demonstrated preferences for a UV-absorbing white and a yellow stimulus, whilst *Lasioglossum* (*Parasphecodes*) sp. showed no colour preferences (Howard et al. 2021). In addition, both achromatic green contrast and spectral purity had a significant positive relationship with the number of visits to stimuli by *L. lanarium* bees (Howard et al. 2021).

In general, intensity is assumed to play a minor role in colour choice by bees (Daumer 1956; Backhaus 1991; Spaethe et al. 2001; Reser et al. 2012; Ng et al. 2018; van der Kooi et al. 2019). A recent study by van der Kooi and Kelber (2022) has shown that for nocturnal and diurnal hawkmoths the intensity of target stimuli plays a role for its visibility. Intensity has been considered as a potentially important factor for flower evolution (Hopkins and Rausher 2012; Renoult et al. 2014; Sletvold et al. 2016), and an experimental approach by Hempel de Ibarra et al. (2000) demonstrated that high brightness contrast between a stimulus and its background can impact the choice behaviour of bees. In bees, achromatic perception of targets is driven by green contrast (modulation of green receptor against the background) and this factor is considered to play an important role in shape processing and motion perception in these insects (von Hess 1913; Kaiser and Liske 1974; Lehrer and Bischof 1995; Hempel de Ibarra and Giurfa 2003; Stach et al. 2004; Stojcev et al. 2011; Morawetz et al. 2013). Furthermore, in several studies green contrast influenced choice behaviour of both honey bees and stingless bees (Giurfa et al. 1996, 1997; Dyer et al. 2016a).

In the past two decades, research on stingless bees has received increased interest as crop pollinators (Heard 1999; Amano et al. 2000; Slaa et al. 2000, 2006; Kremen et al. 2002, 2004; Nunes-Silva et al. 2013; Barbosa et al. 2015). Stingless bees are known to pollinate several crops and in some cases the pollination service offered by stingless bees is more efficient than the one performed by honey bees (Cruz et al. 2005; Dos Santos et al. 2009). Nonetheless, whilst honey bees have been researched for over 100 years (Dyer and Arikawa 2014), the available data on stingless bees are still rather sparse (Heard 2016; Hrncir et al. 2016).

Previous studies concerning the visual capabilities of stingless bees mostly investigated colour choice behaviour with regard to known preferences of model organisms like *A. mellifera* and *B. terrestris* (Dyer et al. 2016a; Koethe et al. 2016). The colour discrimination of temperate species (*A. mellifera* and *B. terrestris*) is finer than in pantropical bee species (Meliponini)(Garcia et al. 2017), although analyses of flower spectral signals in temperate and pantropical regions are almost identical (Chittka and Menzel 1992; Arnold et al. 2010; Dyer et al. 2012, 2021; Shrestha et al. 2013, 2014; Bukovac et al. 2017; Tai et al. 2020).

So far, studies analyzing colour vision in bees suggest that temperate and pantropical bee species may share preferences for blue colour hues, and maybe also the spectral purity of colours, as an honest indicator of flowers offering nectar rewards (Menzel 1967; Chittka and Menzel 1992; Kantsa et al. 2017; Koethe et al. 2016), although in Australia nectar rewards did not correlate with any descriptor of insect-pollinated flower colour signalling (Shrestha et al. 2020). In Australia it has recently been shown that biotic pollination has been the main driver of flower colour evolution (Dalrymple et al. 2020), suggesting understanding visual processing of key pollinations is essential to mapping flowering plant fitness. Currently, however, it is unknown the extent to which colour preferences are common for bees around the world in a way that could be a major driver of flower colour.

In the current study, we tested whether the Australian stingless bee (*Tetragonula carbonaria* Smith) and/or the Western honey bee (*A. mellifera* Linnaeus) choose colours according to the colour parameters spectral purity or intensity by employing stimuli sets that had the same hue but differed either in their spectral purity, or intensity. We specifically hypothesise that (i) *A. mellifera* should demonstrate a significant preference for more pure colour stimuli as has been demonstrated previously, (ii*) T. carbonaria* should demonstrate a significant preference for more pure colours if this feature of bee vision might be phylogenetically conserved. The null hypothesis for respective experiments was choices were not significant from chance expectation. We additionally considered in hypothesis (iii) if honey bees show evidence of being able to learn changes in stimuli intensity and, hypothesis (iv) if *T. carbonaria* bees show evidence of being able to learn changes in stimuli intensity. This question was assessed with fine-tuned changes in stimuli given that several recent ecology studies have assumed intensity may be an important factor in flower evolution (Hopkins and Rausher 2012; Renoult et al. 2014; Sletvold et al. 2016). We discuss our findings with respect to how pollinator perception may influence flower colour evolution.

## Material and methods

### Manufacture of colour stimuli

The manufacture of stimuli to manipulate single colour parameters was enabled using artist pigments as described by (Koethe et al. 2016, 2018). The blue artist pigments were blended to determine the hue of the stimuli (Artist Pigments: “Sky Blue”, “Ultramarine Blue”, Art Material International Warenhandelsgesellschaft mbH, Kaltenkirchen, Germany). For the manipulation of colour intensity and spectral purity, white barium sulphate (99% pure, Grüssing GmbH Analytika, Filsum, Germany; “DeiArt Russverkollerung”, Deifel GmbH & Co. KG, Schweinfurt, Germany), black pigment (carbon black), or a mixture of both achromatic powders, was added to the blue blend. The powders were compacted into culture dishes (35 mm in diameter, 10 mm in height) using a custom-build pigment press. Various combinations of colour intensity and spectral purity were fabricated by mixing defined amounts of the blue blend and varying amounts of white, grey and/or black powders.

**Fig. 1:**
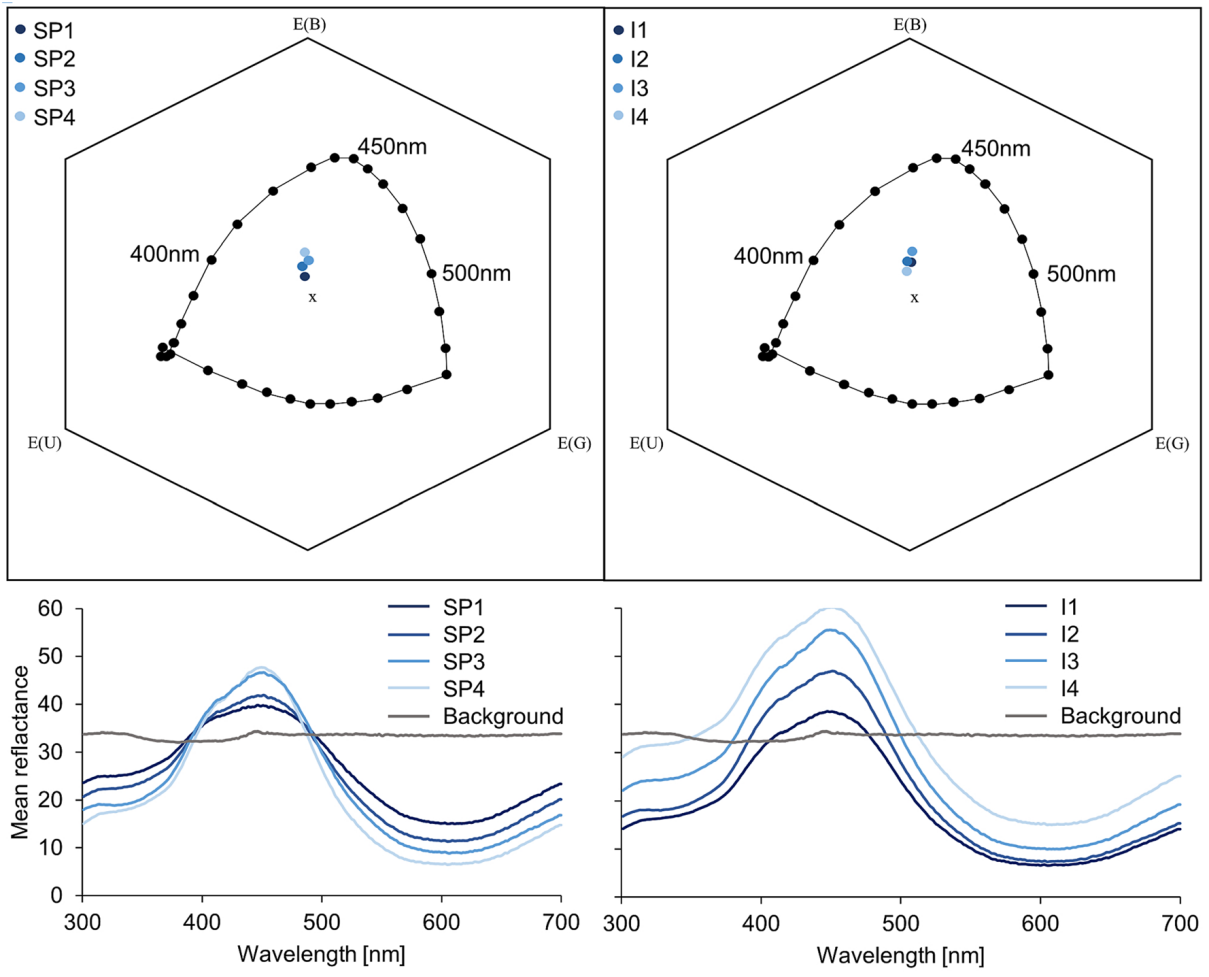
Colour hexagon and reflectance curves of the stimuli. Upper row: The colour hexagon according to Chittka (1992) displays the perception of colours in accordance with bee-specific photoreceptor sensitivities (*Apis mellifera*), the background (grey Styrofoam wallpaper) and the ambient light (standard daylight illumination D65). Lower row: Reflectance curves of all colour stimuli (left: stimuli with manipulated spectral purity SP1 = lowest spectral purity, SP4 = highest spectral purity; right: stimuli with manipulated colour intensity, I1 = lowest intensity, I4 = highest intensity; background = UV-reflecting grey wallpaper)

Spectral reflectance of the produced stimuli was measured using a spectrometer (USB4000 miniature fibre optic spectrometer, Ocean Optics GmbH, Ostfildern, Germany) connected to a UV–NIR deuterium halogen lamp (DH-2000-BAL, Ocean Optics GmbH), through a bifurcated UV–Vis fibre optic cable (Ø 600 µm, QR600-7-UV 125 BX, Ocean Optics GmbH). Readings were recorded at an angle of 45° relative to the horizontal plane. The spectrometer was calibrated against a 2.00% reflectance dark standard (black PTFE powder, Spectralon diffuse reflectance standard SRS-02-010,, Labsphere, Inc. North Sutton, USA) and a 99.0% reflectance white standard (white PTFE powder, Spectralon diffuse reflectance standard SRS99-010, reflectance factor of 99.00%, Labsphere, Inc. North Sutton, USA) (Fig. 1). The obtained spectral data were represented in the colour hexagon model by Chittka (1992) which has been successfully used for both *A. mellifera* and *T. carbonaria* studies (Spaethe et al. 2014; Dyer et al. 2016b; Garcia et al. 2017). Since photoreceptor sensitivities for *T. carbonaria* have not been analysed yet, photoreceptor sensitivities of *Trigona spinipes*, another species of stingless bees (Meliponini) have been used as suggested by previous studies in colour perception in stingless bees (Spaethe et al. 2014; Dyer et al. 2016b). The values calculated for *A. mellifera* and *T. spinipes* are nearly identical (Online Resource 1) and therefore, the values are assumed to be reliable for *T. carbonaria*. The spectral purity was calculated from the perceptual distance between the locus of a colour stimulus and the locus of the centre of the hexagon in relation to the perceptual distance between the locus of the spectral line of the corresponding dominant wavelength colour stimulus and the locus of the centre of the hexagon. ${\text{SP}} = \frac{{H_{i} \left( {{\text{target}} - {\text{background}}} \right)}}{{H_{i} \left( {{\text{spectral locus}} - {\text{background}}} \right)}}$ (Rohde et al. 2013) (Fig. 1). Intensity was calculated by adding up the receptor excitation values for all three photoreceptors and dividing those by the number of photoreceptors [*I* = (*E*_UV_ + *E*_B_ + *E*_G_)/3] (Spaethe et al. 2001). Green contrast was given by the photoreceptor excitation of the green receptor minus 0.5 [GC = *E*_G_ – 0.5] (Spaethe et al. 2001).

Based on the results of these calculations four stimuli with differing levels of spectral purity (SP1 = lowest spectral purity, SP4 = highest spectral purity) but the same intensity level, and vice versa (I1 = lowest intensity, I4 = highest intensity), were selected (Online Resource 1, Fig. 1). The hue of all stimuli was the same to thus allow dissecting the influence of other dimensions of colour signals on bee behaviour in a comparative way. Each stimulus was covered by UV-transmitting Plexiglas discs with an indentation in the centre to offer 10 μL sugar solution (30–50%).

### Experimental arena

To test the bees in a controlled surrounding, an arena was constructed by using grey UV-reflecting wallpaper (for reflectance curve see: Fig. 1; Climapor Insulation Wallpaper Graphite, Saarpor, Neunkirchen, Germany). A 50 × 50 cm plywood board was covered with the same wallpaper and a 13.5 cm high circular wall consisting of the same wallpaper with a diameter of 50 cm was constructed. By choosing this height individual free-flying bees had to inspect stimuli from a close range ensuring colour perception (Online Resource 2; Giurfa et al. 1996). Stimuli were positioned randomly in 36 positions resulting by dividing the internal area of the arena in six columns and six rows. Random allocations of the stimuli for the training and tests were obtained by rolling a dice. Repeated identical positions were re-randomised. The resultant minimum distance between the stimuli was 3 cm. To avoid an influence of casted shadows or proximity to the wall the outer edges of the arena were not taken into consideration for the placement of stimuli.

### Conditioning phase—*A. mellifera*

Honey bees were recruited from university-maintained hives located at the Botanical Garden of the Heinrich-Heine University Düsseldorf, Germany. The bees were freely flying and hence flower experienced. Workers of *A. mellifera* were trained to a UV-transmitting Plexiglas von Frisch type gravity feeder placed outside the arena. As the number of bees varied at different times in the day, the concentration of sugar solution was adjusted to attract more (30%) or fewer bees (10%) to enable the experimental work with a constantly small number of honeybees visiting the feeder. After workers returned frequently to the feeder, the stool on which the feeder was positioned was relocated approximately 5 m towards the area where the training and test took place. The area to which the bees were directed was a shady meadow under some trees with constant lighting conditions. Single workers were trained in the arena by using higher concentrated sugar solution (30–50%), depending on the sugar concentration in the feeder. Higher sugar concentration in the arena as compared to that in the feeder ensures motivation of the tested bees.

Each trained bee was marked individually with nail polish on its dorsal abdomen. For the training, four identical stimuli were used (SP1 or SP4; I1 or I4) thus promoting absolute conditioning to participants in the experiment (Dyer and Chittka 2004; Giurfa 2004). After each visit (Online Resource 3E) to a stimulus the Plexiglas disc was replaced by a clean disc. Training of individual *A. mellifera* bees consisted of three to four foraging bouts and in each foraging bout a bee could visit up to four training stimuli (resulting in 8–13 visits per bee per training). Each bee was either trained to the lowest ranked stimulus (*n* = 20) of intensity (I1) /spectral purity (SP1) or to the highest ranked stimulus (*n* = 20) of the referring parameter (I4/SP4) (Online Resource 3B-C). Each worker subsequently participated in two trainings and two tests; however only the first training and testing condition experienced by bees in the respective four groups is considered in the current manuscript to control for pseudo-replication in the statistical analysis. Specifically, half of the worker bees were trained to the lowest ranked stimulus of a parameter (I1 or SP1) and tested successfully these bees were retrained to the highest ranked stimulus of the respective parameter (I4 or SP4) and vice versa. Thus, 20 individuals were trained first to the lowest ranked stimuli (purity or intensity), and 20 individuals were first trained to the highest ranked stimuli so that in total 80 honey bees were trained and tested. If more than one bee returned to the arena all additional bees were captured in tubes and released after finishing the experiment with the first bee. After completing the experiment each worker was sacrificed to avoid pseudo-replication of data. Whilst only the data from a bee’s first conditioning experiment is considered for statistical analyses in the current manuscript to avoid the complexities that bees do not reverse learn colour information as a consistent group (Dyer et al. 2014), all raw collected data is available as supplementary files (Online resource 7).

### Conditioning phase—*T. carbonaria*

Hives of *T. carbonaria*—provided by Sugarbag Bees (sugarbag.net)—were kept in an urban environment in Brisbane, Australia. A gravity feeder made of UV-transmitting Plexiglas was placed in the middle of the arena to attract workers of *T. carbonaria* (Online Resource 3 A)*.* If more than one bee returned to the gravity feeder all additional bees were captured in tubes and released after finishing the experiment with the test bee. The released workers that willingly returned to the arena could subsequently be trained and tested; marking was thus not necessary. Workers of T. *carbonaria* are much smaller (Online Resource 3F) than workers of *A. mellifera* and typically imbibe less than 5 μL of sucrose solution per visit (Norgate et al. 2010), and therefore visited only one stimulus per foraging bout. Each worker of *T. carbonaria* was trained for eight foraging bouts to ensure a training effect comparable to that observed in honey bees. As with *A. mellifera* bees, four identical stimuli were used for training. Two training approaches per parameter were also used for *T. carbonaria* with either the lowest ranked stimulus (I1 or SP1) or the highest ranked stimulus (I4 or SP4) like in honey bees. A total of 80 workers of *T. carbonaria* were tested in our experiments: first training I1 (*n* = 20); first training I4 (*n* = 20); first training SP1 (*n* = 20); first training SP4 (*n* = 20).

After completing the second test each worker was sacrificed to avoid pseudo-replication. For the statistical analyses only the first conditioning experienced was analysed to address the research hypotheses of the current manuscript. All raw data is made available via supplementary files (Online Resource 7).

### Test phase

The experiments were designed to enable comparisons. Each bee was tested for either intensity or spectral purity and each bee completed two rounds of testing (see above); however, only the first five choices of each bee was evaluated. A total of 40 workers of each species were tested for variations in intensity—based on trainings starting with high ranked stimuli (*n* = 20) and trainings starting with low ranked stimuli (*n* = 20)—and another 40 workers of each species were tested for variations in spectral purity—based on trainings starting with high ranked stimuli (*n* = 20) and trainings starting with low ranked stimuli (*n* = 20), resulting in 80 workers per species in total. In each test eight stimuli were presented (two stimuli per level) that offered water instead of a sugar solution reward and five decisions per bee of this unrewarding test were recorded and evaluated (Online Resource 3 D). The first choices of the stingless bees and honeybees are given in Online Resources 5 and 6.

### Statistical analyses

To formally test if there are differences in the frequency of choices made by *Apis mellifera* and *Tetragonula carbonaria* to stimuli of similar colour but differing intensity or purity, we constructed four multinomial baseline category logit models (Agresti 2013). Each one of these models allowed us to test for the effect of species, the independent factor, on changes in the log odds of selecting stimuli SP2, SP3, and SP4 relative to SP1 which was set as the baseline for all comparisons. We included a random term on each of our multinomial models to account for individual variability. The random term imposes a correlation structure to all choices made by a single individual (Zuur et al. 2013), thus accounting for the variability resulting from the repeated measures experimental design.

Log odds are relative measurements of probability and their magnitude is interpreted as a factor of increment, or reduction, on the success of a given event (Faraway 2006); thus, large positive values of a log odds ratio can be interpreted as an increase in the likelihood of an event to occur. For our model, we can interpret a positive log odd ratio as an increase in the chances of a bee visiting a stimulus of a higher signal relative to the baseline by a factor equal to the magnitude of the ratio. Log odds are also a measurement of effect size in linear models using the logit transformation (Field 2009).

Models were fitted using the mclogit package (Elff 2021) for the R statistical language. The effect of species in the model was tested using a likelihood ratio (LRT) following standard methods for testing logit linear models (Faraway 2006).

## Results

### Model 1: purity trained to low signal stimulus

**Fig. 2:**
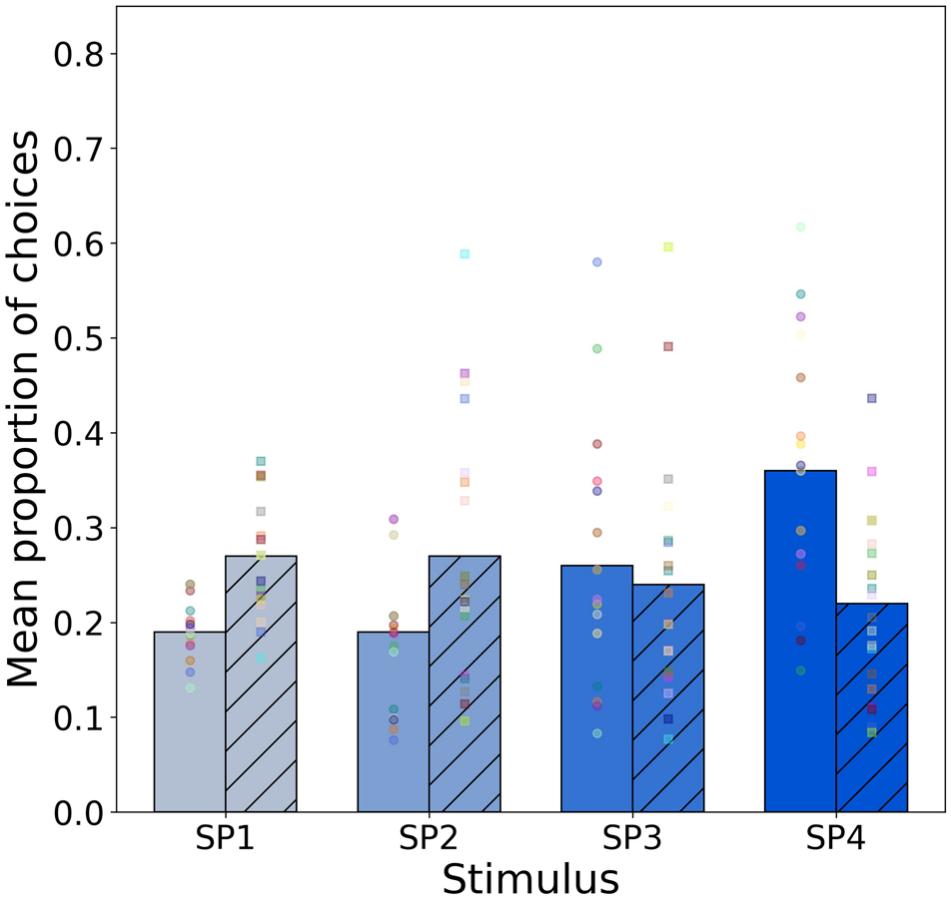
Mean proportion of choices for purity stimulus SP1-SP4 for *A. mellifera* (solid bars) and *T. carbonaria* (hatched bars) following conditioning to the stimulus of the lowest spectral purity*.* Mean proportion values were predicted from the multinomial model fitting the observed data. Markers on each bar represent the frequency of choices made to each stimulus by 20 individual bees considering the first five choices each. Circle markers *A. mellifera*, square markers *T. carbonaria*

Model 1 considered the responses of independent groups of *A. mellifera* (*n* = 20) and *T. carbonaria* bees (*n* = 20), factor species with two levels, to colour stimuli of constant intensity, but varying in spectral purity, when the initial priming conditioning was to the stimulus of the lowest spectral purity. For this experiment, the responses (Fig. 2) for either bee species were not significantly different from chance expectation (*χ*^2^ = 2.45, *df* = 3, *P* = 0.485) and there was no evidence for a significant change in the odds of selecting a stimulus of higher spectral purity for all considered pairs (Table 1).

**Table 1:** Predicted log odds ratio for the three purity stimuli pairs for European honey bees and stingless bees trained to a low signal stimulus

Stimuli pair	*A. mellifera* log odds (95% confidence intervals)	*T. carbonaria* log odds (95% confidence intervals)
SP2/SP1	− 0.083 (− 0.903, 0.737)	− 0.094 (− 2.02, 1.84)
SP3/SP1	0.218 (− 0.537, 0.973)	− 0.214 (− 2.02, 1.59)
SP4/SP1	0.588 (− 0.119, 1.30)	− 0.281 (− 2.00, 1.44)

Asterisks indicate the significance of the change in log odds for any given pair: * *P* < 0.05, ** *P* < 0.01 and *** *P* < 0.001. Model coefficients and respective *z*-values are reported in Online Resource 4 provided as supplementary material

### Model 2: purity trained to high signal stimulus

**Fig. 3:**
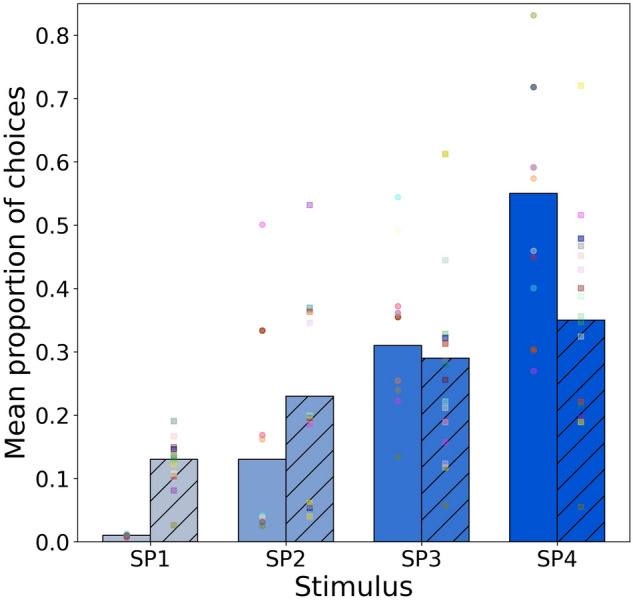
Mean proportion of choices for purity stimulus SP1-SP4 for *A. mellifera* (solid bars) and *T. carbonaria* (hatched bars) following conditioning to the stimulus of the highest spectral purity. Mean proportion values were predicted from the multinomial model fitting the observed data. Markers on each bar represent the frequency of choices made to each stimulus by 20 individual bees after 5 trials each. Circle markers *A. mellifera*, square markers *T. carbonaria*

Model 2 considered the responses of independent groups of *A. mellifera* (*n* = 20) and *T. carbonaria* (*n* = 20) bees to colour stimuli of constant intensity but varying in spectral purity when the initial priming conditioning was to the stimulus of the highest purity (Fig. 3). This model suggests a difference in the change of odds for stimuli of varying spectral purity between species (*χ*^2^ = 12.6, *df* = 3, *P* = 0.005). Given the trend of choices modelled for the two bee species (Fig. 3), the significance of this variable likely arises from a difference in the magnitude of the effect observed for the two species. Specifically, whilst both species show an increase in the odds of selecting the stimulus presenting the highest signal with increasing spectral purity, this effect is larger in honey bees than in stingless bees (Table 2). For example, *Apis* has 4 times more chances of choosing the highest spectral purity stimuli relative to the lowest spectral purity stimuli, whilst the odds of choosing the highest spectral purity stimulus for the same pair increases by a factor of one for *Tetragonula.* This result thus suggests that whilst both species are sensitive to changes in spectral purity, and prefer stimuli presenting higher levels of this quality, this preference is more pronounced in honey bees than in stingless bees.

**Table 2:** Predicted log odds ratio for the three spectral purity stimuli pairs for European honey bees and stingless bees trained to a high signal stimulus

Stimuli pair	*A. mellifera* log odds (95% confidence intervals)	*T. carbonaria* log odds (95% confidence intervals)
SP2/SP1	1.97 (− 0.361, 4.3)	0.216 (− 4.741, 5.21)
SP3/SP1	3.40 (1.37, 5.43)**	0.726 (− 3.47, 4.93)*
SP4/SP1	3.96 (1.95, 5.96)***	0.934 (− 3.2, 5.07)**

Asterisks indicate the significance of the change in log odds for any given pair: * *P* < 0.05, ** *P* < 0.01 and *** *P* < 0.001. Model coefficients and respective *z*-values are reported in Online Resource 4 provided as supplementary material

### Model 3: intensity trained to a low signal stimulus

**Fig. 4:**
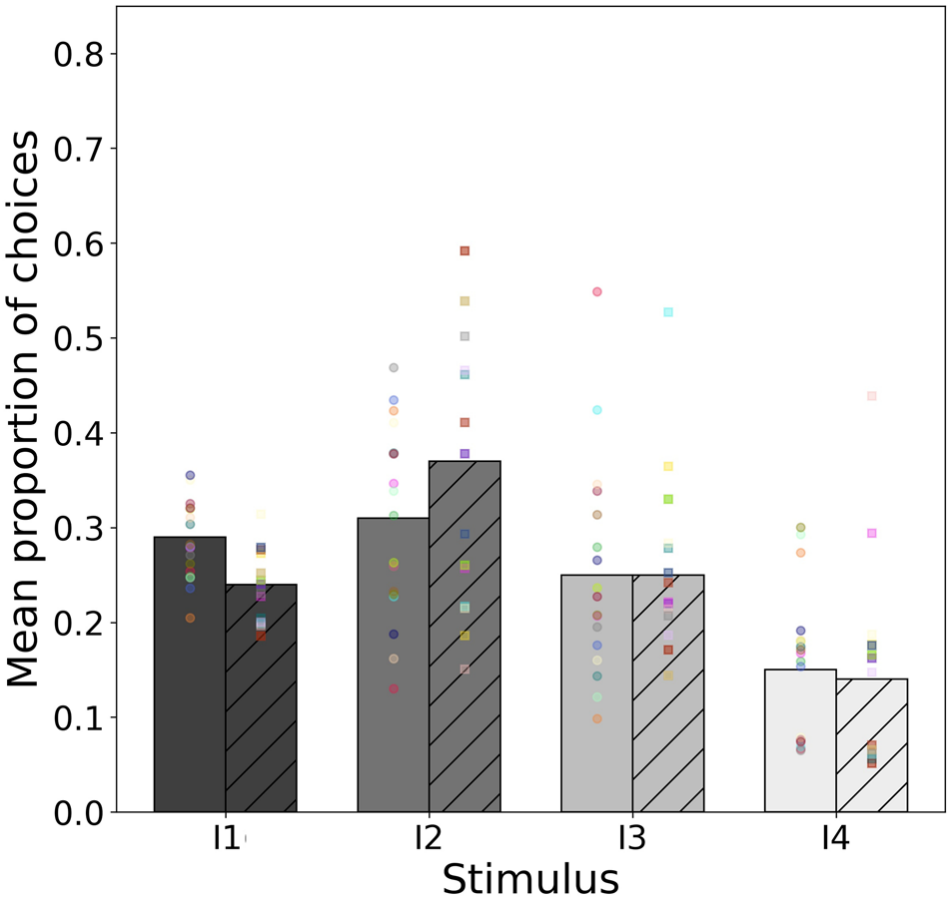
Mean proportion of choices for intensity stimulus I1-I4 for *A. mellifera* (solid bars) and *T. carbonaria* (hatched bars) following conditioning to the stimulus of the lowest intensity. Mean proportion values were predicted from the multinomial model fitting the observed data. Markers on each bar represent the frequency of choices made to each stimulus by 20 individual bees after 5 trials each. Circle markers *A. mellifera*, square markers *T. carbonaria*

Model 3 considered the responses (Fig. 4) by independent groups of *A. mellifera* (*n* = 20) and *T. carbonaria* bees (*n* = 20) for similar colour stimuli that differed in intensity when the initial priming conditioning was to the stimulus of the lowest intensity. For this experiment, the choices for either bee species were not significantly different from chance expectation (*χ*^2^ = 0.582, *df* = 3, *P* = 0.900) and there was no evidence for a significant change in the odds of selecting a stimulus of higher intensity for all considered pairs (Table 3).

**Table 3:** Predicted log odds ratio for the three intensity stimuli pairs for European honey bees and stingless bees trained to a low signal stimulus

Stimuli pair	*A. mellifera* log odds (95% confidence intervals)	*T. carbonaria* log odds (95% confidence intervals)
I2/I1	0.022 (− 0.621, 0.664)	0.369 (− 1.19, 1.93)
I3/I1	− 0.228 (− 0.889, 0.434)	0.001 (− 1.61, 1.61)
I4/I1	− 0.781 (− 1.665, 0.104)	− 0.728 (− 2.89, 1.436)

Asterisks indicate the significance of the change in log odds for any given pair: * *P* < 0.05, ** *P* < 0.01 and *** *P* < 0.001. Model coefficients and respective *z*-values are reported in Online Resource 4 provided as supplementary material

### Model 4: intensity trained to high signal stimulus

**Fig. 5:**
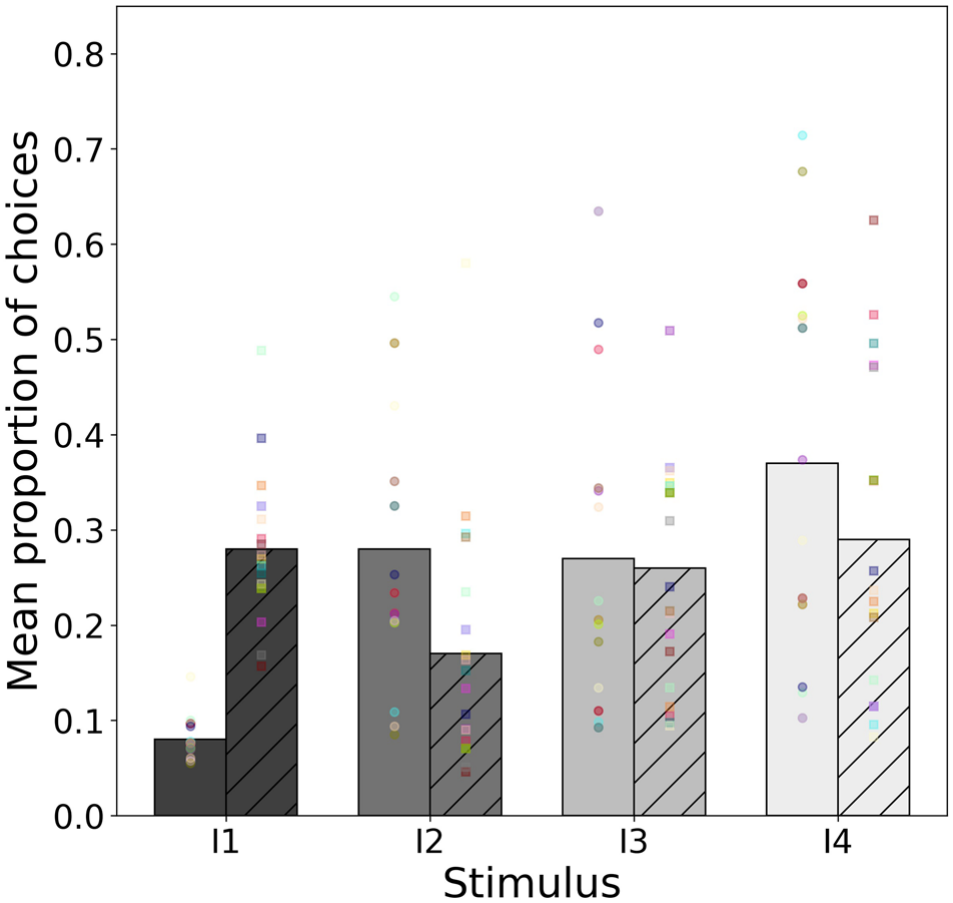
Mean proportion of choices for intensity stimulus I1-I4 for *A. mellifera* (solid bars) and *T. carbonaria* (hatched bars) following conditioning to the stimulus of the highest intensity. Mean proportion values were predicted from the multinomial model fitting the observed data. Markers on each bar represent the frequency of choices made to each stimulus by 20 individual bees after 5 trials each. Circle markers *A. mellifera*, square markers *T. carbonaria*

Model 4 considered the responses (Fig. 5) by independent groups of *A. mellifera* (*n* = 20) and *T. carbonaria* (*n* = 20) bees for similar colour stimuli that differed in intensity, when the initial priming conditioning was to the stimulus of the highest intensity. For this experiment we found a significant difference in the changes of odds ratios with stimuli between species (*χ*^2^ = 11.9, *df* = 3, *P* = 0.008). This difference arises by the higher odds of choosing the stimulus with higher intensity of each pair by *A. mellifera* compared to *T. carbonaria* (Table 4). Thus, the odds of choosing a stimulus with higher intensity does show evidence of increasing with stimulus magnitude in honey bees. On the other hand, the model indicates that *T. carbonaria* bees do not lower the odds of choosing the stimulus of the lowest intensity. Overall the comparative analyses indicate some sensitivity in *A. mellifera* to intensity when other factors like hue differences are tightly controlled, and absolute conditioning to a more intense stimulus is used.

**Table 4:** Predicted log odds ratio for the three intensity stimuli pairs for European honey bees and stingless bees trained to a high signal stimulus

Stimuli pair	*A. mellifera* log odds (95% confidence intervals)	*T. carbonaria* log odds (95% confidence intervals)
I2/I1	1.16 (0.213, 2.11) *	− 0.69 (− 2.90, 1.51)**
I3/I1	1.04 (0.039, 2.04) *	− 0.16 (− 2.44, 2.12)
I4/I1	1.38 (0.416, 2.35)**	− 0.120 (− 2.32, 2.11)*

Asterisks indicate the significance of the change in log odds for any given pair: * *P* < 0.05, ** *P* < 0.01 and *** *P* < 0.001. Model coefficients and respective *z*-values are reported in Online Resource 4 provided as supplementary material

## Discussion

There is currently considerable interest in plant-pollinator interactions (Dyer et al. 2019; Paulus 2019; van der Kooi and Stavenga 2019) for which bees are often the most important pollinators (Chittka and Menzel 1992; Dyer et al. 2012). However, comparative studies of how different bee species perceive the different dimensions of colour stimuli are relatively few (Howard 2021; Howard et al. 2021). The current study considered colour preferences of both honey bees and stingless bees considering perceptually similar colour stimuli, and analogous testing procedures.

Considering our first hypothesis, for Western honey bees we observed that colour choices were significantly influenced by the spectral purity of tested stimuli when receiving absolute conditioning to the stimulus of highest spectral purity as rewarding (Fig. 3). However, when independent honey bees were conditioned towards the stimulus of lowest spectral purity (Fig. 2) there was no significant preference towards any stimuli. These results are consistent with previous findings that honey bees that are conditioned to specific stimuli do still also demonstrate a preference for more spectrally pure colours (Papiorek et al. 2013; Rohde et al. 2013), but also show that such a preference is also plastic and can be modulated suggesting that plants must provide honest signalling to receive flower constant continuous visits. Thus plant flowers may receive some benefit of getting first visits by bees if displaying more spectrally pure colours, but such visits will not remain preferentially towards these colours unless there is a reward.

The values of spectral purity of the tested stimuli correlate with the values of colour contrast between background and target colour (Online resource 1). Spectral purity is defined as the perceptual distance of a target colour from the uncoloured locus (Chittka and Kevan 2005), whereas colour contrast is defined as the perceptual distance between any two colours. Recently, van der Kooi and Spaethe (2022) pointed to this kind of correlation between values of spectral purity and colour contrast in several studies predominantly of flower colours. In our study, the spectral purity is calculated as the hexagon distance of a colour locus from the locus of the background colour in relation to the hexagon distance between the locus of the background colour and the corresponding spectral locus as proposed by Rhode et al. (2013) and the colour contrast is calculated as the hexagon distance between a colour locus and the background locus. The experimental setting used in this study, comprising only four stimuli of similar dominant wavelength, is not eligible to distinguish between responses of the tested bees to spectral purity and colour contrast as was done by Lunau et al. (1996) using variation of background and test colours and colour pattern of artificial stimuli.

Considering our second hypothesis we also observed evidence that stingless sugarbag bees significantly preferred more spectrally pure colours only when conditioned to more spectrally pure stimuli (Figs. 2, 3), although their observed preference for the pure colour had a smaller effect than that observed in Western honey bees (Table 2). This difference in effect size between species likely underpins why previous research with dissimilar colour stimuli did not observe that sugarbag bees prefer purity as a sole factor, as with different broadband colour stimuli multiple factors of colour be confounded potentially masking weaker results where a combination of purity and green contrast was significantly correlated with choices, but green contrast in isolation was a much stronger predictor of behaviour (Dyer et al. 2016a). Thus, consistent with other recent work on bee colour perception (Koethe et al. 2016, 2018), multiple factors influence how colours are chosen and stimuli require careful design to dissect mechanisms underpinning choices. Taken together, the results of the current study, and also the previous work on bumblebees (Papiorek et al. 2013; Rohde et al. 2013) do suggest that honest signalling with more spectrally pure colours may be a key factor in why plant flowers frequently have such saturated colours as perceived by human observers. According to Exner and Exner (1910) “we can discern manifold designs in flower petals which generate a relatively high colour saturation. Thus we must … conclude that the more saturated colours are more conspicuous than unsaturated ones for insects … as it is the case for us. Only the most brilliantly coloured jewels surpass certain flower colours in colour saturation” (translated from German by the authors).

A second main research question considered in the current study was whether either species of bees tested might prefer stimulus intensity when colour hue and spectral purity was controlled. This question is important as several recent ecology studies on flower signalling have suggested that in some cases the brightness or intensity of a flower may be a factor in attracting pollinators (Hopkins and Rausher 2012; Renoult et al. 2014; Sletvold et al. 2016; van der Kooi and Kelber 2022). In honey bees we thus tested hypothesis (iii) and the results show that variations in stimulus intensity do significantly influence preferences when bees were conditioned towards the most intense colour (Fig. 5), however, when independent honey bees were conditioned towards the least intense colour then no significant preference was evidenced for any stimulus in resulting tests (Fig. 4). Hypothesis (iv) tested if sugarbag bees demonstrated any preference for stimulus intensity where observed differences were marginal or in the opposite direction to that expected from conditioning protocol (Figs. 4, 5; Tables 3, 4), suggesting intensity is not a biologically important cue in these bees. In a recent study, Ng et al. (2018) tested the choice behaviour of honey bees to detect bee-achromatic stimuli based on intensity when presented at a visual angle to promote colour processing. The honey bees failed to detect the stimuli, although they were very accurate at detecting control stimuli containing chromatic contrast. These findings that honeybees did not show a significant preference considering stimulus intensity is consistent with several previous studies on free-flying honey bees (Daumer 1956; Backhaus 1991; Reser et al. 2012). Thus the evidence in the current study that only honey bees, and only in one condition show evidence of processing intensity differences of colour even when absolute conditioning is provided (Fig. 5; Table 4) suggests that intensity is indeed a difficult cue for bees to process, although there may be some plasticity of the visual system to acquire this dimension of perception when absolute conditioning is used and other masking factors are excluded.

In previous studies, stingless bees showed significant preferences for blueish colour hues considering stimuli with high colour contrasts (Dyer et al. 2016a; Koethe et al. 2016). In these studies, only *Partamona helleri* showed a significant preference for spectrally purer colours when choosing stimuli with the same colour hue (Koethe et al. 2018). In several tropical bees a preference for colour stimuli dominated by short wavelengths and those dominated by long wavelengths was found (Balamurali et al. 2018). In a study conducted in a Mediterranean scrubland, the amount of nectar and the degree of spectral purity positively correlated and hence provide a possible explanation for a preference of more spectrally pure colours in flower visitors like found in honey bees (Kantsa et al. 2017). However, in Australia there was not significant relationship between the nectar content of flowers and their colour appearance (Shrestha et al. 2020), thus suggesting there may be regional differences in how different bee preferences may affect local communities. Indeed the relatively weak preference of sugarbag bees towards pure colours as compared to honey bees (Table 2) is a plausible explanation, although there is a need to test more bee species in more environments to understand how such effects might influence flower colouration at a localised level.

In conclusion, colour preferences among the two tested bee species show some similarities in that there is evidence of a spontaneous preference towards more spectrally pure colours, since training to high spectral purity resulted in a clear preference for stimuli of high spectral purity, whereas low spectral purity stimuli did not result in a preference for stimuli of low spectral purity. The differences in that effect were much stronger in Western honey bees. Our analysis suggests that when intensity is the only factor of colour considered, the probability of choosing a stimulus significantly increases with intensity in honeybees, although this effect is small. On the other hand, the probability of choosing stimuli is independent of the intensity in sugarbag bees when tested under the same conditions. Our comparative findings on two bee species, *Apis mellifera* and *Tetragonula carbonaria*, that evolved in different environments, suggests that colour processing differences including spectral purity or stimulus intensity do exist. It is thus important to test, where possible, the psychophysics of biologically relevant animals when evaluating the potential effects of colour decision-making on visual ecology processes.

## Supplementary Information

Below is the link to the electronic supplementary material.

Supplementary file1 (PDF 460 KB)

Supplementary file2 (PDF 171 KB)

Supplementary file3 (PDF 443 KB)

Supplementary file4 (PDF 364 KB)

Supplementary file5 (PDF 197 KB)

Supplementary file6 (PDF 196 KB)

Supplementary file7 (XLSX 24 KB)
