# Supplementary material for: Comparative psychophysics of Western honey bee (Apis mellifera) and stingless bee (Tetragonula carbonaria) colour purity and intensity perception
Source: J Comp Physiol A Neuroethol Sens Neural Behav Physiol. 2022 Oct 21;208(5-6):641–52. doi: 10.1007/s00359-022-01581-y (PMC9734212; doi:10.1007/s00359-022-01581-y)
Supplement: Supplementary file 1 — Supplementary file1 (PDF 460 KB) [file 359_2022_1581_MOESM1_ESM.pdf]

Online Resource 1 Calculated colour parameters of stimuli

Apis mellifera	I1	I2	I3	I4	SP1	SP2	SP3	SP4
**UV receptor**	0.3791	0.4104	0.4683	0.5219	0.4581	0.4412	0.4182	0.4037
**Blue receptor**	0.4983	0.5440	0.5852	0.6127	0.5235	0.5293	0.5451	0.5433
**Greenreceptor**	0.3617	0.3992	0.4467	0.4954	0.4421	0.4226	0.4156	0.3890
**Colour contrast**	0.1288	0.1395	0.1291	0.1065	0.0748	0.0987	0.1282	0.1475
**Green contrast**	-0.1383	-0.1008	-0.0533	-0.0046	-0.0579	-0.0774	-0.0844	-0.1110
**Blue contrast**	-0.0017	0.0440	0.0852	0.1127	0.0235	0.0293	0.0451	0.0433
**Brightness contrast**	1.2391	1.3536	1.5002	1.6300	1.4236	1.3931	1.3789	1.3360
**Spectral purity**	0.2734	0.2955	0.2857	0.2459	0.1694	0.2177	0.2721	0.3154
**Intensity**	0.4130	0.4512	0.5001	0.5433	0.4745	0.4644	0.4596	0.4453
Trigona spinipes	**I1**	**I2**	**I3**	**I4**	**SP1**	**SP2**	**SP3**	**SP4**
**UV receptor**	0.3750	0.4046	0.4643	0.5199	0.4569	0.4389	0.4400	0.3979
**Blue receptor**	0.4962	0.5416	0.5833	0.6109	0.5224	0.5278	0.5794	0.5409
**Green receptor**	0.3416	0.3774	0.4255	0.4776	0.4300	0.4068	0.4311	0.3658
**Colour contrast**	0.1410	0.1524	0.1424	0.1180	0.0823	0.1086	0.1440	0.1614
**Green contrast**	-0.1584	-0.1226	-0.0745	-0.0224	-0.0700	-0.0932	-0.0689	-0.1342
**Blue contrast**	-0.0038	0.0416	0.0833	0.1109	0.0224	0.0278	0.0794	0.0409
**Brightness contrast**	1.2128	1.3235	1.4731	1.6084	1.4093	1.3735	1.4506	1.3046
**Spectral purity**	0.2807	0.2966	0.2946	0.2545	0.1731	0.2308	0.2807	0.3333
**Intensity**	0.4043	0.4412	0.4910	0.5361	0.4698	0.4578	0.4835	0.4349
