# Supplementary material for: Comparative psychophysics of Western honey bee (Apis mellifera) and stingless bee (Tetragonula carbonaria) colour purity and intensity perception
Source: J Comp Physiol A Neuroethol Sens Neural Behav Physiol. 2022 Oct 21;208(5-6):641–52. doi: 10.1007/s00359-022-01581-y (PMC9734212; doi:10.1007/s00359-022-01581-y)
Supplement: Supplementary file 2 — Supplementary file2 (PDF 171 KB) [file 359_2022_1581_MOESM2_ESM.pdf]

Online Resource 2 Calculation of arena wall height

In order to calculate the height of the arena's wall necessary to enable colour vision and disable sole perception via green contrast the following equation was applied:
$h = g/(2*\tan(\alpha/2))$. (h = height of the arena; g = diameter of stimulus; α = visual angle)(Giurfa et al. 1996).

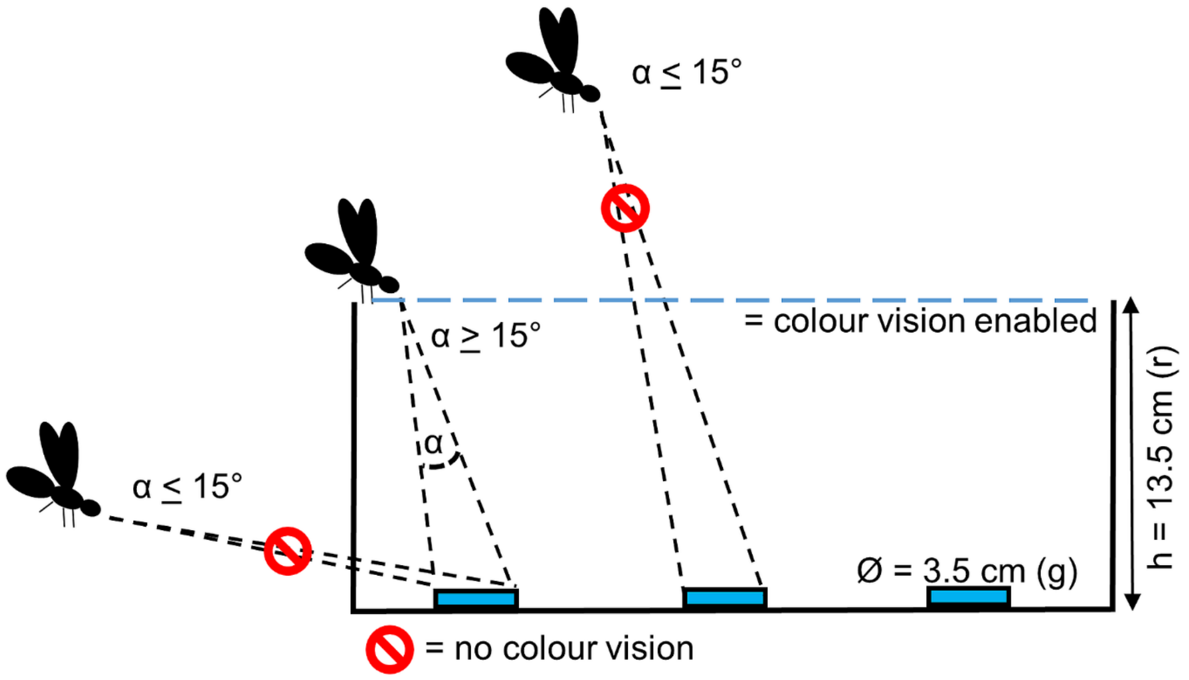

Giurfa M, Vorobyev M, Kevan P, Menzel R (1996) Detection of coloured stimuli by honeybees: minimum visual angles and receptor specific contrasts. J Comp Physiol A 178: 699–709.
