# Supplementary material for: Comparative psychophysics of Western honey bee (Apis mellifera) and stingless bee (Tetragonula carbonaria) colour purity and intensity perception
Source: J Comp Physiol A Neuroethol Sens Neural Behav Physiol. 2022 Oct 21;208(5-6):641–52. doi: 10.1007/s00359-022-01581-y (PMC9734212; doi:10.1007/s00359-022-01581-y)

Online Resource 3 Experimental setup

(A) Testing arena with an UV-transmitting Feeder in its centre. (B-C) Training situations (as an example) for high and low spectral purity that were conducted consecutively separated by a test run. The same procedure was conducted for the stimuli with manipulated intensity. (D) Exemplary test arrangement with eight stimuli (two of each stimulus SP1-SP4 or I1-I4). (E-F) Foraging worker of *A. mellifera* and *T. carbonaria* on a stimulus.

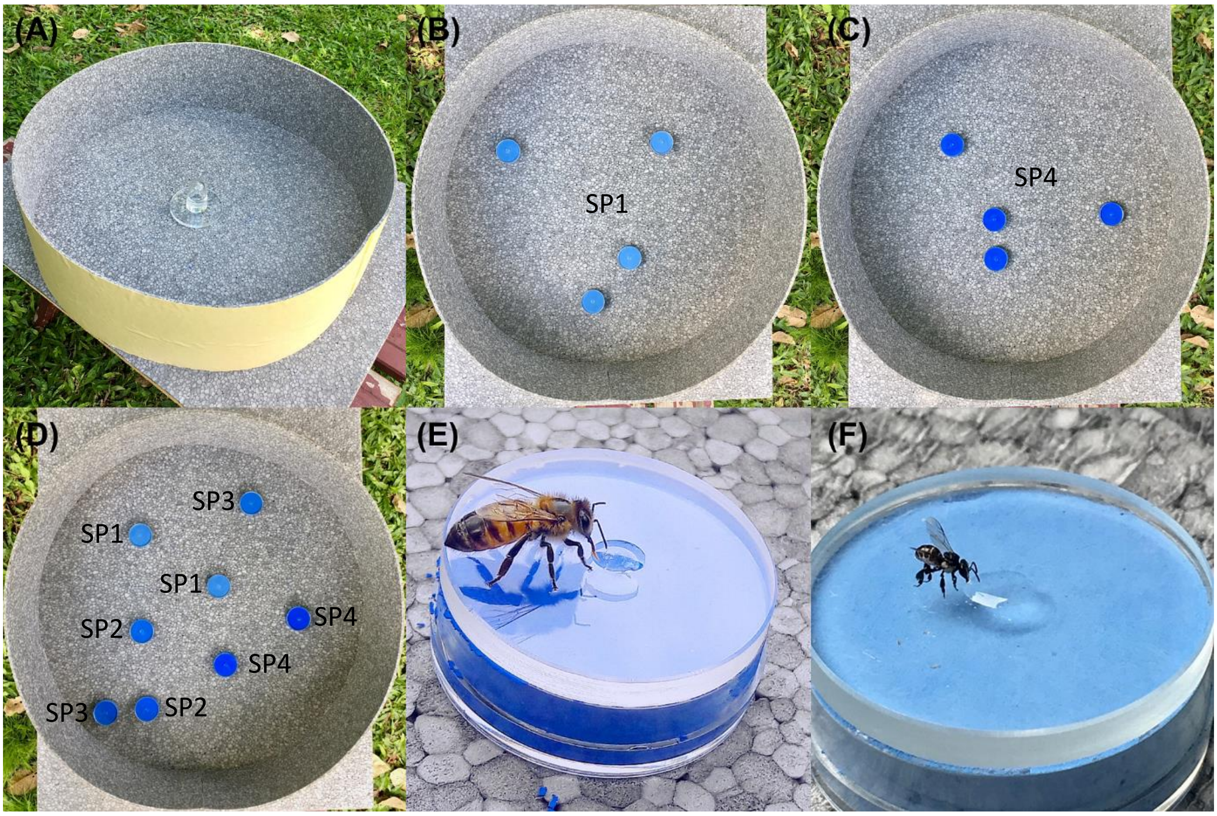

Supplement: Supplementary file 3 — Supplementary file3 (PDF 443 KB) [file 359_2022_1581_MOESM3_ESM.pdf]
