# Supplementary material for: Comparative psychophysics of Western honey bee (Apis mellifera) and stingless bee (Tetragonula carbonaria) colour purity and intensity perception
Source: J Comp Physiol A Neuroethol Sens Neural Behav Physiol. 2022 Oct 21;208(5-6):641–52. doi: 10.1007/s00359-022-01581-y (PMC9734212; doi:10.1007/s00359-022-01581-y)
Supplement: Supplementary file 4 — Supplementary file4 (PDF 364 KB) [file 359_2022_1581_MOESM4_ESM.pdf]

Online Resource 4 Results of Z-transformation according to Fisher

T1 = first training; T2 = second training; rho = Pearson's correlation coefficient; min. = lower limit of confidence interval; max. = upper limit of the confidence interval

	T1	rho	min.	max.	T2	rho	min.	max.	*p*-value
T. carbonaria	SP1	0.9234	1.1361	2.0869	SP1	-0.9487	-2.2938	-1.3431	<0.001
	SP4	0.9909	2.2169	3.1676	SP4	0.7746	0.5563	1.5071	<0.001
	I1	0.8889	0.9416	1.8923	I1	-0.5758	-1.1316	-0.1808	<0.001
	I4	0.2828	-0.1846	0.7661	I4	0.7379	0.4704	1.4211	0.028
A. mellifera	SP1	0.9311	1.1915	2.1422	SP1	0.8627	0.8286	1.7793	0.145
	SP4	0.9891	2.1266	3.0774	SP4	0.9932	2.3668	3.3175	0.242
	I1	-0.8706	-1.8108	-0.8601	I1	-0.8581	-1.7615	-.08108	0.443
	I4	0.9106	1.0555	2.0062	I4	0.8367	0.7346	1.6853	0.175
