# Supplementary figures and images for: Comparative psychophysics of Western honey bee (Apis mellifera) and stingless bee (Tetragonula carbonaria) colour purity and intensity perception
Source: J Comp Physiol A Neuroethol Sens Neural Behav Physiol. 2022 Oct 21;208(5-6):641–52. doi: 10.1007/s00359-022-01581-y (PMC9734212; doi:10.1007/s00359-022-01581-y)

## Online Resource 5 First choices of *T. carbonaria*

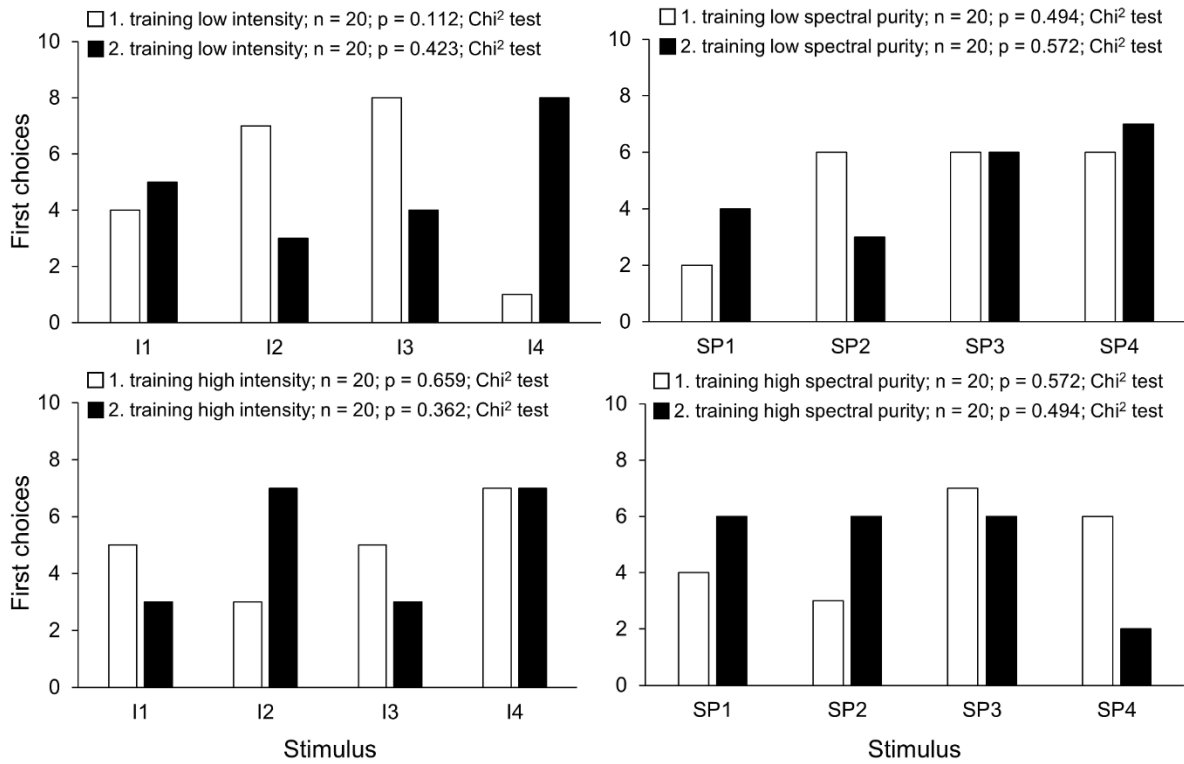

Supplement: Supplementary file 5 — Supplementary file5 (PDF 197 KB) [file 359_2022_1581_MOESM5_ESM.pdf]

# 1 Online Resource 6 First choices of *A. mellifera*

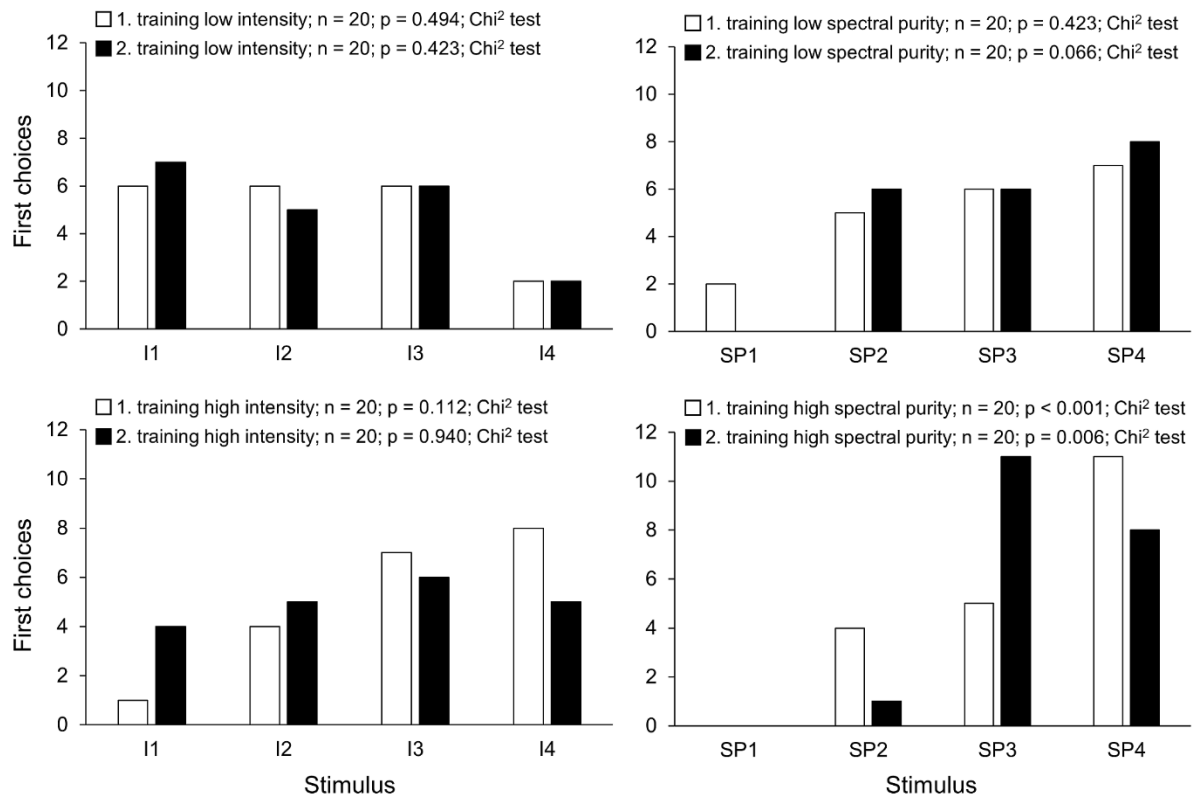

2

3

4

Supplement: Supplementary file 6 — Supplementary file6 (PDF 196 KB) [file 359_2022_1581_MOESM6_ESM.pdf]
